# Basal-Forebrain Cholinergic Nuclei Alterations are Associated With Medication and Cognitive Deficits Across the Schizophrenia Spectrum

**DOI:** 10.1093/schbul/sbad118

**Published:** 2023-08-22

**Authors:** Julia Schulz, Felix Brandl, Michel J Grothe, Matthias Kirschner, Stefan Kaiser, André Schmidt, Stefan Borgwardt, Josef Priller, Christian Sorg, Mihai Avram

**Affiliations:** TUM-NIC Neuroimaging Center, Technical University of Munich, School of Medicine, Munich, Germany; Department of Neuroradiology, Technical University of Munich, School of Medicine, Munich, Germany; TUM-NIC Neuroimaging Center, Technical University of Munich, School of Medicine, Munich, Germany; Department of Neuroradiology, Technical University of Munich, School of Medicine, Munich, Germany; Department of Psychiatry and Psychotherapy, Technical University of Munich, School of Medicine, Munich, Germany; Unidad de Trastornos del Movimiento, Servicio de Neurología y Neurofisiología Clínica, Instituto de Biomedicina de Sevilla, Hospital Universitario Virgen del Rocío/CSIC/Universidad de Sevilla, Sevilla, Spain; Department of Psychiatry, Psychotherapy and Psychosomatics, Psychiatric Hospital, University of Zurich, Zurich, Switzerland; Department of Psychiatry, Geneva University Hospital, Geneva, Switzerland; Department of Psychiatry (UPK), University of Basel, Basel, Switzerland; Translational Psychiatry, Department of Psychiatry and Psychotherapy, University of Lübeck, Lübeck, Germany; Department of Psychiatry and Psychotherapy, Technical University of Munich, School of Medicine, Munich, Germany; TUM-NIC Neuroimaging Center, Technical University of Munich, School of Medicine, Munich, Germany; Department of Neuroradiology, Technical University of Munich, School of Medicine, Munich, Germany; Department of Psychiatry and Psychotherapy, Technical University of Munich, School of Medicine, Munich, Germany; Translational Psychiatry, Department of Psychiatry and Psychotherapy, University of Lübeck, Lübeck, Germany

**Keywords:** magnetic resonance imaging, first-episode psychosis, cholinergic system, cognitive difficulties

## Abstract

**Background and Hypothesis:**

The cholinergic system is altered in schizophrenia. Particularly, patients’ volumes of basal-forebrain cholinergic nuclei (BFCN) are lower and correlated with attentional deficits. It is unclear, however, if and how BFCN changes and their link to cognitive symptoms extend across the schizophrenia spectrum, including individuals with at-risk mental state for psychosis (ARMS) or during first psychotic episode (FEP).

**Study Design:**

To address this question, we assessed voxel-based morphometry (VBM) of structural magnetic resonance imaging data of anterior and posterior BFCN subclusters as well as symptom ratings, including cognitive, positive, and negative symptoms, in a large multi-site dataset (*n* = 4) comprising 68 ARMS subjects, 98 FEP patients (27 unmedicated and 71 medicated), 140 patients with established schizophrenia (SCZ; medicated), and 169 healthy controls.

**Results:**

In SCZ, we found *lower* VBM measures for the *anterior* BFCN, which were associated with the anticholinergic burden of medication and correlated with patients’ cognitive deficits. In contrast, we found larger VBM measures for the *posterior* BFCN in FEP, which were driven by unmedicated patients and correlated at-trend with cognitive deficits. We found no BFCN changes in ARMS. Altered VBM measures were not correlated with positive or negative symptoms.

**Conclusions:**

*Results* demonstrate complex (posterior vs. anterior BFCN) and non-linear (larger vs. lower VBM) differences in BFCN across the schizophrenia spectrum, which are specifically associated both with medication, including its anticholinergic burden, and cognitive symptoms. Data suggest an altered trajectory of BFCN integrity in schizophrenia, influenced by medication and relevant for cognitive symptoms.

## Introduction

Schizophrenia is a debilitating psychiatric disorder with a lifetime prevalence of approximately 1%.^1^ Its symptoms are categorized into 3 clusters: Psychotic (eg, hallucinations and delusions), negative (eg, anhedonia and social withdrawal), and cognitive (eg, deficits in executive function and attention).^1^ While the disorder is pathognomonically primarily defined by psychotic symptoms, negative and cognitive symptoms account for a large proportion of everyday dysfunction in the long term.^2–4^ Although patients are typically first diagnosed in early adulthood with occurrence of the first psychotic episode, the disorder’s onset of negative and cognitive symptoms is suggested to begin earlier, consistent with the aberrant underdevelopment characterization of the disorder.^1,5^ The at-risk phase is characterized by cognitive and functional impairments and social withdrawal, with these symptoms potentially exacerbating and leading to a first psychotic episode.^1,3^ Psychotic symptoms fluctuate over the course of schizophrenia (ie, psychosis and psychotic remission), while negative and cognitive symptoms persist or worsen over time.^6,7^ Both the distinct characteristics of the disorder and their heterogeneous course have led to the concept of “schizophrenia spectrum,” which includes at-risk mental state for psychosis (ARMS), first psychotic episode (FEP), and established schizophrenia (SCZ).^8^

Schizophrenia is heterogeneous not only regarding its symptoms and their course but also at the level of underpinning genetics and pathophysiological pathways, including abnormalities in neuromodulatory systems.^9^ Beyond well-known alterations of the dopaminergic pathway (ie, the dopamine hypothesis of schizophrenia^10,11^), the cholinergic system seems to be particularly impaired.^12,13^ The main neurotransmitter of the cholinergic system is acetylcholine (ACh), which mediates signaling through its binding affinity for two receptor families, muscarinic and nicotinic. Both receptor types are distributed throughout the brain, and their subtypes are localized pre- and postsynaptically.^14,15^ ACh release throughout the brain is mediated by both widely distributed cholinergic interneurons and cholinergic projecting neurons, which are mostly located in the brainstem cholinergic nuclei and the basal-forebrain cholinergic nuclei (BFCN).^16^ The BFCN neurons project to cortical and subcortical structures following a distinct topographical organization and contribute to basic cognitive functions, including attention and working memory.^14,16,17^ Cytoarchitectonically, BFCN neurons are divided into 4 main cell groups; furthermore, by the use of functional connectivity analyses, the human BFCN can be divided into an anterior part—including medial septal nucleus, diagonal band of Broca, and anterior-medial parts of the nucleus basalis of Meynert that project to the hippocampus, ventromedial prefrontal, and retrosplenial/posterior cingulate cortex,—and a posterior part—comprising the remaining parts of the nucleus basalis of Meynert, which project to the rest of the brain including amygdala nuclei.^18,19^

Mounting evidence suggests that several aspects of the cholinergic system are altered in schizophrenia.^12^ Altered cholinergic transmission has been reported both by postmortem findings of reduced numbers of ACh receptors,^20,21^ and in vivo imaging studies, using single-photon emission computed tomography or positron emission tomography, demonstrating decreased availability of ACh receptors in schizophrenia.^22–25^ Furthermore, pharmacological manipulation of the cholinergic system in patients demonstrated improved memory and attention and enhanced activation of multiple brain regions involved in cognitive functioning.^26^ Treatment with a muscarinic cholinergic receptor agonist improved not only patients’ positive symptoms but also negative symptoms and their global clinical impression.^27^ Recently, we demonstrated that the structural integrity of the whole BFCN cluster is altered in SCZ.^28^ Specifically, we found that magnetic resonance imaging (MRI)-based voxel-based morphometry (VBM) measures of BFCN were decreased in two independent samples of patients.^28^ It remains, however, elusive both when the BFCN alterations appear in the course of schizophrenia and to what extent they depend on the stage of the disorder, including chronicity and long-term treatment. For instance, there is evidence that early cholinergic alterations are relevant for psychosis and its onset.^12,29^ To address this question of occurring BFCN changes along the schizophrenia course indirectly (ie, using a cross-sectional design), the current study focuses on the BFCN subdivisions’ structure and its alterations across the stages of the schizophrenia spectrum. Due to the relevance of cholinergic changes for psychotic states, we hypothesized that VBM of both anterior and posterior BFCN are not only altered in SCZ but also in earlier stages.

Next, regarding the relevance of these structural changes for schizophrenia symptoms, we previously found that in SCZ, lower BFCN-VBM mediates patients’ attentional deficits, measured by the Symbol-coding Task.^28^ This result is consistent both with previous studies demonstrating a link between aberrant cholinergic transmission and cognitive deficits in schizophrenia as well as animal and human studies demonstrating the general relevance of the cholinergic system for cognitive functioning.^12^ In particular, animal studies have shown that the structural integrity of the BFCN and their projections are crucial for several cognitive functions.^30^ Human evidence, based on in vivo neuroimaging, demonstrated that BFCN structural integrity links with cognition in healthy subjects.^31^ While structural changes in the cholinergic system are relevant for cognitive deficits in schizophrenia, it is unclear whether a similar link exists in other stages of the schizophrenia spectrum. We hypothesized that structural alterations occur across stages of the spectrum, with potential changes in both anterior and posterior BFCN being associated with patients’ cognitive deficits.

To address these hypotheses, we studied T1-weighted MRI and performance on cognitive tests from cross-sectional multi-site data of the schizophrenia spectrum, including ARMS, FEP, SCZ, and healthy controls (HC).^28,32–37^ 94 SCZ and 97 HC of this dataset overlap with our previous study on BFCN volume in SCZ.^28^ Furthermore, we used the same VBM approach as previously,^28^ namely VBM. These measures of BFCN were derived from T1-weighted images by combining volumetry with a stereotactically deﬁned BFCN atlas.^38–40^

## Material and Methods

### Participants

We studied a multi-site dataset comprising 68 ARMS, 98 FEP (27 unmedicated, 71 medicated), 140 SCZ (medicated), and 169 HC (overview: table 1).

**Table 1.** Participants, Datasets, Clinical Symptom Scales, and Tests. Names of Groups, Scales, and Tests are Provided With Their Abbreviations as Used Throughout the Text

Datasets								
	HC	ARMS	No Transition	Transition	FEP	Unmedicated	Medicated	SCZ
Munich	24							24
COBRE	73							70
Zurich	28				26	-	26	46
Basel	44	68	53	15	72	27	45	
**total**	**169**	**68**	53	15	**98**	27	71	**140**
								
*Clinical and cognitive ratings*								
	**PANSS**	**SANS**	**TMT-A**	**SCT**	**PF**	**MWT-B**		
Munich	x		x	x		x		
COBRE	x		x	x	x			
Zurich	x				x	x		
Basel	x	x			x	x		

*Note*: ARMS individuals with a high risk for psychosis, FEP patients with first-episode psychosis, HC healthy controls, MWT-B Multiple-Choice vocabulary intelligence test Part B, PANSS Positive and Negative Syndrom Scale, PF Phonemic Fluency, TMT-A Trail Making Test Part A, SANS Scale for the Assessment of Negative Symptoms, SCT Symbol-coding Task, SCZ patients with schizophrenia

#### Munich Dataset.

The sample was acquired by our group in Munich^28,36,37^ and included 26 SCZ along with 24 age- and sex-matched HC. Diagnosis of SCZ was confirmed using the Structured Clinical Interview for DSM-IV.^41^

#### COBRE Dataset.

Seventy-two SCZ meeting DSM-IV criteria,^41^ and 73 HC from the open-source Center for Biomedical Research Excellence (COBRE) database (http://fcon_1000.projects.nitrc.org/indi/retro/cobre.html) were included.

#### Zurich Dataset.

Data of 48 SCZ, 26 FEP, and 28 age- and sex-matched HC were obtained from previous publications.^32,33,42^ Diagnosis of SCZ and FEP was determined with the Mini-International Neuropsychiatric Interview for DSM-IV.

#### Basel Dataset.

The sample of 78 FEP, 73 ARMS, and 44 HC was derived from previous studies.^34,35^ Diagnosis of FEP was determined by the Brief Psychiatric Rating Scale as described by Yung and colleagues.^43,44^ ARMS was confirmed based on Personal Assessment and Crisis Evaluation criteria.^44^

All studies were approved by the local ethics committees, and participants provided written informed consent.

### Assessment of Cognitive Deficits

To assess cognitive abilities, the Trail Making Test Part A (TMT-A) or Phonemic Fluency test was used. We used two different test batteries as the four sites employed different cognitive testing, and therefore, not all tests were available for all patient groups. TMT-A mainly measures processing speed and visual attention and is reported as seconds required to solve the task.^45^ Phonemic Fluency provides information on processing speed and semantic memory based on retrieval of words beginning with a specific letter.^46^

For demographic and clinical scores, see table 2.

**Table 2.** Demographic And Clinical Variables Across the Schizophrenia Spectrum. Data are Presented as Means and Standard Deviations. Names of Groups, Scales, and Tests are Provided With Their Abbreviations as Used Throughout the Text

Complete (All Studies, *n* = 475)	HC (*n* = 169)	ARMS (*n* = 68)	FEP (*n* = 98)	SCZ (*n* = 140)	*P*-value
Age [years]	32.89 (±10.76)	24.68 (±5.16)	26.67 (±7.33)	37.36 (±12.35)	<.001^*^^,^^1^
Sex (f/m)	69/100	19/49	25/73	34/106	.006^*^^,^^2^
Illness duration [years]				13.90 (±10.75)	N/A^1^
CPZ equivalents [mg/d]			210.20 (±249.82)	459.52 (±378.17)	<.001^*^
ACB score [a.u.]			1.57 (±1.41)	2.75 (±1.70)	<.001^*^^,^^1^
					
COBRE, Munich, Zurich			FEP (*n* = 26)	SCZ (*n* = 140)	
PANSS positive [a.u.]			5.96 (±1.80)	11.58 (±5.41)	<.001^*^^,^^1^
					
COBRE, Munich, Zurich				SCZ (*n* = 140)	
PANSS negative [a.u.]				14.17 (±5.32)	N/A^1^
					
Basel			FEP (*n* = 43)		
SANS [a.u.]			24.78 (±16.83)		N/A^1^
					
COBRE, Munich	HC (*n* = 88)			SCZ (*n* = 86)	
TMT-A [sec]	23.59 (±7.53)			39.80 (±15.37)	<.001^*^^,^^1^
					
COBRE, Munich	HC (*n* = 89)			SCZ (*n* = 90)	
SCT [a.u.]	62.00(±10.70)			43.39 (±11.74)	<.001^*^^,^^1^
					
COBRE, Zurich, Basel	HC (*n* = 94)	ARMS (*n* = 25)	FEP (*n* = 37)	SCZ (*n* = 98)	
PF [a.u.]	24.70 (±5.55)	22.65 (±5.86)	19.14 (±7.52)	15.35 (±7.67)	<.001^*^^,^^1^
					
Munich, Zurich, Basel	HC (*n* = 46)	ARMS (*n* = 47)	FEP (*n* = 67)	SCZ (*n* = 23)	
MWT-B [a.u.]	28.43 (±4.57)	29.38 (±3.47)	26.27 (±5.82)	29.39 (±3.61)	.28 ^1^

^1^Mean differences tested with ANOVA.

^2^Mean differences tested with chi-squared test.

^*^Statistical signiﬁcance at *P* < .05.

### Image Acquisition and Processing

MRI acquisition parameters are described in supplementary methods.

**Fig. 1.**
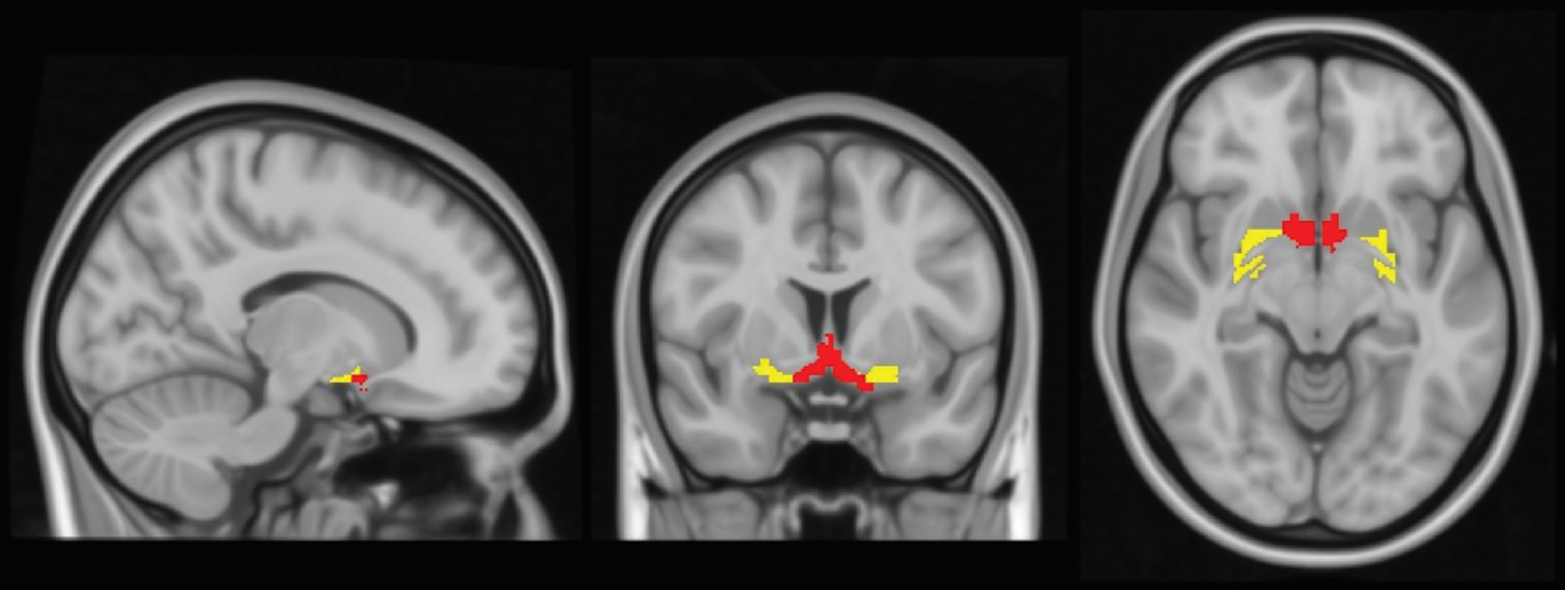
Anterior and posterior basal-forebrain cholinergic nuclei (BFCN). The colored ROI shows the BFCN overlaid on a human brain template. The mask was derived from cytoarchitectonic mappings based on combined histology and postmortem magnetic resonance imaging reflecting a consensus of all available stereotactic BFCN masks.^38–40^ The anterior subcluster (red, center) represents the medial septal nucleus, diagonal band, and anterior-medial parts of the nucleus basalis of Meynert (NBM), whereas the posterior part (yellow, left and right) corresponds to the remaining part of the NBM.^18^

T1-weighted structural images from the 4 datasets were processed separately using CAT12 (http://dbm.neuro.uni-jena.de/cat/) and SPM12,^47^ implemented in MATLAB R2019b (The MathWorks, Inc., Natick, MA). Default parameters for preprocessing were used following the standard protocol (https://neuro-jena.github.io/cat/) unless otherwise stated. Specifically, images were segmented into gray matter (GM), white matter, and cerebral spinal fluid partitions of 1.5-mm isotropic voxel size. Each participant’s partitions in native space were registered to a stereotactic standard space (ie, Montreal Neurological Institute space). To preserve the total amount of GM within each voxel, the partitioned images were modulated by the relative voxel values (ie, the Jacobian determinants) before and after normalization. The resulting GM-VBM was then smoothed with a Gaussian kernel full-width-half-maximum of 4mm due to the small size of the region-of-interests and in line with our previous approach (Supplemental,^28^). To control segmentation and registration accuracy, GM maps were visually inspected. Briefly, in this study, 5 ARMS (from the Basel cohort), 6 FEP (from the Basel cohort), and 6 SCZ patients (2 from the COBRE, 2 from the Munich, and 2 from the Zurich cohort) were excluded based on quality control and missing demographic information. Quality control in this study was performed based on interquartile range (IQR) output from SPM12. Images with an IQR of 5 standard derivations below and above the mean IQR were excluded. Modulated GM voxel values within a ROI encompassing the BFCN were summed to extract individual GM-VBM of the anterior and posterior BFCN, respectively, from the warped GM segments. The BFCN-ROI is based on a consensus of currently available stereotactic BFCN masks. The masks were derived from cytoarchitectonic mappings based on histology and postmortem MRI.^19,38–40^ The anterior subcluster includes the medial septal nucleus, diagonal band, and anterior-medial parts of the nucleus basalis Meynert, and the posterior subcluster contains the remaining part of the nucleus basalis Meynert (figure 1).^18^ This mask was also used in our prior article in a control analysis to differentiate the anterior and posterior subclusters.^28^ Total intracranial volume (TIV) was used to normalize the individual BFCN- and GM-VBM for differences in head size, as previously described.^48^

### Statistical Analysis

To enable comparison between the four acquisition sites, VBM were harmonized using the open-source toolbox neuroCombat 0.2.12 implemented in Python 3.7 (https://github.com/Jfortin1/neuroCombat). NeuroCombat adjusts data for scanner effects by using a linear regression model combined with empirical Bayes.^49^

Analyses were performed on TIV-normalized VBM of all four combined datasets (*n* = 475). Statistics were carried out using Python 3.10.6 and SPSS 23.0 (IBM Corporation, Somers, NY).

*Group differences in BFCN-VBM*. Differences between groups (HC, ARMS, FEP, and SCZ) and subgroups (HC, FEP-unmedicated, and FEP-medicated) in anterior and posterior BFCN and global GM-VBM were evaluated using univariate analysis of covariance (ANCOVA), controlling for the influence of age and sex. For subsequent post hoc analyses, Dunnett’s test with HC as control group was used.

Control and specificity analyses were performed for effects of antipsychotic medication (chlorpromazine equivalents, CPZ), anticholinergic burden (ACB) of medication, total brain volume, age, and illness duration (see supplementary material for details).

*Associations between altered BFCN-VBM and cognitive deficits.* Differences between groups in cognitive performance were tested using ANCOVA. Depending on the distribution of the data, Pearson’s and Spearman’s correlation analysis was used to investigate the relationship between patients’ changed BFCN-VBM and cognitive symptoms. Subjects with missing cognitive scores were excluded.

## Results

### Anterior BFCN-VBM is Lower in Patients With Schizophrenia

**Fig. 2.**
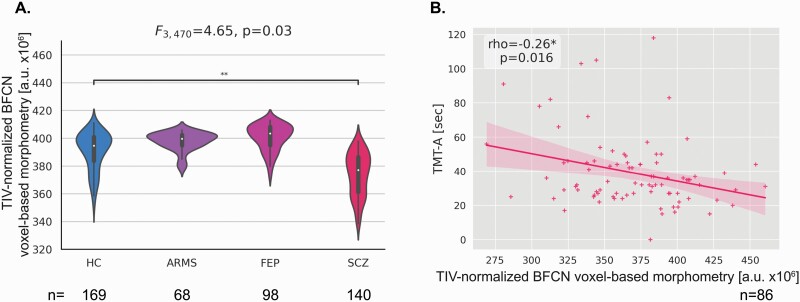
Lower anterior basal-forebrain cholinergic nuclei (BFCN)-VBM in schizophrenia links with cognitive impairment. (A) Group comparisons between HC, ARMS, FEP, and SCZ regarding anterior BFCN-VBM were investigated using ANCOVA controlling for age and sex. Dunnett’s was used for post hoc analysis. *<0.05, **<0.01, and ***<0.001. Anterior BFCN-VBM differed significantly across the schizophrenia spectrum (F_3,470_ = 4.65, *P* = .03) and was lower in SCZ compared to HC (*P* = .003). (B) Spearman correlation analysis revealed a signiﬁcant correlation between anterior BFCN-VBM and TMT-A (rho = −0.26, *P* = .016). ARMS individuals with a high risk for psychosis, BFCN Basal-forebrain cholinergic nuclei, FEP patients with first-episode psychosis, HC healthy controls, SCZ patients with schizophrenia, TMT-A Trail Making Test Part A, VBM, voxel-based morphometry.

Group comparison across the schizophrenia spectrum revealed a significant difference in TIV-normalized anterior BFCN-VBM as demonstrated by an ANCOVA, using sex and age as covariates (F_3,470_ = 4.65, *P* = .03). Anterior BFCN-VBM was lower in SCZ compared to HC (*P* = .003, figure 2A).

Several control and specificity analyses were conducted (for details, see supplementary results and figure S1). Briefly, we found that the group comparison only remained at-trend toward significance when additionally controlling for ACB. Correlation analysis showed that the anticholinergic burden of medication was linked to anterior BFCN-VBM in SCZ (rho = −0.22, *P* = .009), suggesting that a higher level of anticholinergic burden of medication is relevant for lower anterior BFCN measures in SCZ. Antipsychotic medication in terms of chlorpromazine equivalents (CPZ) did not correlate with anterior BFCN-VBM. Next, global GM-VBM changes affected our ANCOVA result, which remained only at-trend significant when controlling for global GM-VBM. The association between lower anterior BFCN-VBM and ACB is also not independent of global GM-VBM, indicated by partial correlation analysis. Although age correlated with anterior BFCN-VBM in both HC and SCZ, we did not find an interaction between age and group, indicating that the disorder’s effect on BFCN was not further increased by age. Illness duration did not influence the group differences in anterior BFCN-VBM.

### Posterior BFCN-VBM is Larger in Patients With First-Episode Psychosis

**Fig. 3.**
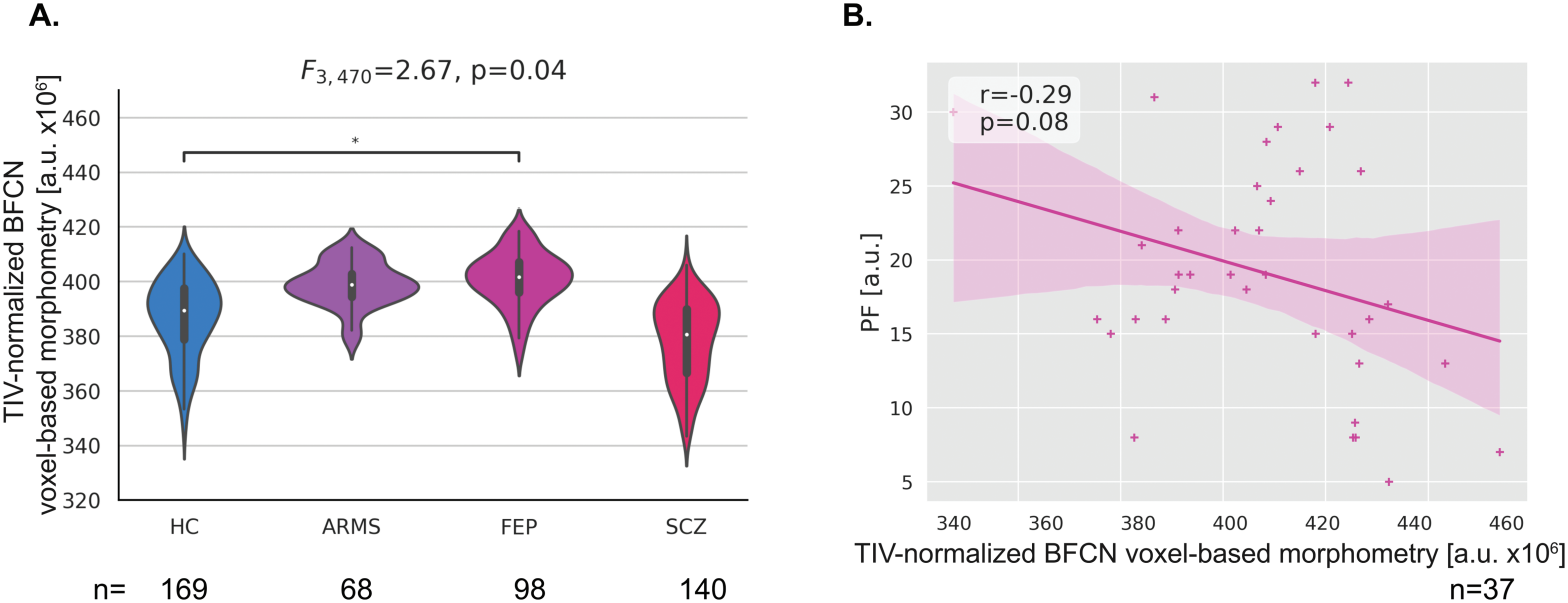
Larger posterior basal-forebrain cholinergic nuclei (BFCN)-voxel-based morphometry (VBM) in first-episode psychosis links at-trend with cognitive impairment. (A) Group comparisons between HC, ARMS, FEP, and SCZ regarding posterior BFCN-VBM were investigated using ANCOVA controlling for age and sex. Dunnett’s was used for post hoc analysis. *<0.05, **<0.01, and ***<0.001. Posterior BFCN-VBM differed significantly across the schizophrenia spectrum (F_3,470_ = 2.67, *P* = .04) and was larger in FEP compared to HC (*P* = .05). (B) Pearson correlation analysis revealed a correlation at-trend between posterior BFCN-VBM and PF (*r* = −0.29, *P* = .08). ARMS individuals with a high risk for psychosis, BFCN Basal-forebrain cholinergic nuclei, FEP patients with first-episode psychosis, HC healthy controls, PF Phonemic Fluency, SCZ patients with schizophrenia, VBM, voxel-based morphometry.

ANCOVA, using sex and age as covariates, showed a significant difference in posterior BFCN-VBM across the schizophrenia spectrum (F_3,470_ = 4.94, *P* = 0.002). Posterior BFCN-VBM was larger in FEP compared to HC (*P* = .05, figure 3A).

Several control and specificity analyses were conducted (for details, see supplementary results and figure S2). Briefly, subgroup analysis showed larger posterior BFCN-VBM in FEP-unmedicated compared to HC (*P* < .001), indicating that larger posterior BFCN-VBM were mainly driven by FEP-unmedicated. Group differences in posterior BFCN-VBM only remained at-trend significant after controlling for global GM. However, it should be noted that we did not find differences in global GM-VBM between HC and FEP. Nonetheless, we cannot exclude that the overall GM-VBM influences the differences in posterior BFCN-VBM in FEP.

### In Schizophrenia, Lower Anterior BFCN-VBM Correlates With Impaired Cognition

To investigate whether lower anterior BFCN-VBM in SCZ was linked to processing speed deficits, measured by TMT-A, both ANOVA and subsequent correlation analysis were performed (figure 2B). First, we found significantly worse performance on TMT-A in SCZ, indicating cognitive deficits (F_172,1_ = 42.15, *P* < .001). A significant negative correlation was found between lower anterior BFCN-VBM in SCZ and TMT-A (rho = −0.26, *P* = .016), indicating that lower anterior BFCN-VBM is associated with more severe cognitive deficits. We found the same correlation between anterior BFCN-VBM and other cognitive tests (ie, Symbol-coding Task), suggesting the independence of this relationship from the cognitive test used. To investigate whether additional variables such as ACB, sex, and age influenced the relationship between lower anterior BFCN-VBM and TMT-A scores, we computed a multiple regression analysis (see supplementary results). Results indicate that anterior BFCN-VBM significantly predicts TMT-A scores as long as age is not included in the model, as age and anterior BFCN-VBM were highly collinear (rho = −0.44, *P* < .001).

Specificity analyses were conducted to control the influence of other factors (for details, see supplementary results and figure S3). Briefly, lower anterior BFCN-VBM was not correlated with negative and positive symptoms, respectively, suggesting a symptom-specific link between anterior BFCN and cognitive symptoms in SCZ. Anterior BFCN-VBM was not correlated with cognitive performance in HC, suggesting disorder-specificity for the link between anterior BFCN and cognitive symptoms. Our finding was not influenced by anticholinergic-burden of medication or the used cognitive test.

### In First-Episode Psychosis, Larger Posterior BFCN-VBM Correlates At-trend With Impaired Cognition

Focusing on the clinical relevance of posterior BFCN-VBM difference in FEP, we studied the association between larger posterior BFCN-VBM and deficits in processing speed, measured by Phonemic Fluency, with both ANOVA and correlation analysis. We found significantly reduced Phonemic Fluency performance in FEP (F_129,1_ =21.67, *P* < .001), indicating cognitive deficits. Furthermore, we identified a negative at-trend-to-significance correlation between larger posterior BFCN-VBM and Phonemic Fluency (*r* = −0.29, *P* = .08; figure 3B). This at-trend result may indicate that larger BFCN-VBM contributes to cognitive impairments in FEP. However, this interpretation warrants caution, given that we conducted analyses in several groups.

Specificity and control analyses were performed (for details, see supplementary results and figure S4). Briefly, we found the same at-trend correlation between posterior BFCN-VBM and other cognitive tests (ie, Multiple-choice vocabulary intelligence test Part B), suggesting both the independence of this relationship from the cognitive test used and confirming that posterior BFCN changes link with cognitive deficits in FEP. Interestingly, we found anticholinergic-burden to influence the association in FEP-medicated. Furthermore, larger posterior BFCN-VBM was not correlated with negative and positive symptoms, respectively, suggesting a symptom-specific link between posterior BFCN and cognitive symptoms in FEP. Posterior BFCN-VBM was not correlated with cognitive performance in HC, suggesting a disorder-specific link.

## Discussion

By the use of T1-weighted MRI VBM of cytoarchitectonically defined regions of the anterior and posterior BFCN and cognitive assessment, we found both distinct changes in BFCN subclusters across the schizophrenia spectrum and specific associations between these changes and cognitive deficits: The anterior BFCN-VBM was lower in SCZ, and patients’ VBM was associated with the anticholinergic-burden of medication and impaired processing speed; the posterior BFCN-VBM was larger in FEP, with larger individual VBM in unmedicated patients and linked at-trend to impaired processing speed. To the best of our knowledge, this is the first study extending findings of alterations in the cholinergic system, namely BFCN structure, from schizophrenia to the schizophrenia spectrum. Our findings demonstrate complex (posterior vs. anterior BFCN) and non-linear (larger vs. lower VBM) differences in BFCN across the schizophrenia spectrum, which are influenced by both medications, including its anticholinergic-burden, and consistently and specifically associated with cognitive symptoms. Data suggest an altered, medication-influenced, cognitive symptom-relevant trajectory of BFCN integrity in schizophrenia.

### Lower Anterior BFCN-VBM in Schizophrenia

We found significantly lower anterior BFCN-VBM in SCZ compared to HC and FEP. This is in line both with previous structural imaging findings reporting lower VBM across the whole and anterior BFCN in SCZ^28^ and postmortem and single-photon emission computed tomography/PET studies demonstrating altered synaptic cholinergic transmission in SCZ.^20–23,25,50^

We interpret these differences in BFCN-VBM as reflecting volume reductions in anterior BFCN. MRI-based volumetric measurements are not a direct measure of brain structure. Changes in cells, cell number, water content, tissue perfusion, and both artifacts and confounders, such as head motion and smoking, contribute to the T1-weighted MRI signal.^51^ Lipid in myelin and membranes is also suggested to be crucial for the MRI contrast by shortening the relaxation time.^52^ In SCZ, lipid composition appears disturbed, and this disturbance might be further influenced by antipsychotic medication.^53–56^ Conversely, postmortem findings revealed overall reduced GM volume, especially in the limbic system and thalamus, and decreased cell density in schizophrenia.^57,58^ Therefore, the observed differences in anterior BFCN MRI-signal might result from a combination of decreased lipid levels due to both long-term treatment with antipsychotics and actual reduction in GM tissue.

The group difference in anterior BFCN-VBM also seems to be influenced by anticholinergic-burden of medication. This suggests that the level of anticholinergic-burden of medication might be relevant for changes in the cholinergic system in SCZ, indicating a link between lower BFCN-VBM and cholinergic transmission. We found lower global GM-VBM in SCZ—a result consistently reported by previous neuroimaging studies.^59–61^ Group differences in anterior BFCN-VBM changed from highly significant to at-trend toward significant after controlling for global GM-VBM. Therefore, we cannot exclude that anterior BFCN-VBM decrease is parallel with general GM-VBM decrease in SCZ. Interestingly, ACB was associated with GM-VBM, indicating that while the lower anterior BFCN-VBM is not independent of global GM-VBM, both measures are influenced by the anticholinergic burden of medication in SCZ. Anterior BFCN-VBM further correlated with age in both HC and SCZ, indicating that age influences anterior BFCN-VBM. Moderation analysis showed that age did not interact with the disorder's effect on anterior BFCN-VBM, suggesting that the disorder's influence on anterior BFCN is not modified by age. Furthermore, we found neither a main effect of illness duration nor an interaction effect of age and illness duration on anterior BFCN-VBM. These findings suggest that BFCN-VBM is not altered in SCZ as a function of illness duration. This is in contrast to global GM-VBM reductions, which become more pronounced with advancing illness duration.^62,63^

We found no anterior BFCN-VBM differences in ARMS and FEP compared to HC. While a lack of differences in early stages of the schizophrenia spectrum may indicate that changes only occur later during the illness course, we cannot completely exclude changes in earlier stages. Indeed, the ARMS group examined in this study was quite heterogeneous, and only a part transitioned to psychosis at a later time point. However, a recent multimodal-imaging study demonstrated microstructural alterations of the anterior BFCN in FEP,^64^ suggesting that changes in anterior BFCN are already present in FEP but may only be detected at the volumetric level at a later stage.

### Larger Posterior BFCN-VBM in FEP

We found that posterior BFCN-VBM was larger in FEP compared to HC. Particularly, posterior BFCN-VBM was larger in FEP-unmedicated compared to HC. Intriguingly, posterior BFCN-VBM did not differ between controls and FEP-medicated, suggesting that the group differences in posterior BFCN-VBM across the spectrum may be driven by unmedicated patients. Conversely, Park and colleagues found no differences in myelin content and axonal integrity in the posterior BFCN in FEP-unmedicated.^64^ However, the two volumetric and microstructural measures differ, making direct comparisons difficult. Whether antipsychotic medication accounts for changes in VBM not only during FEP but also along the course of schizophrenia continues to be debated. For example, research has provided evidence for associations between GM reductions and higher doses of antipsychotics.^62,63,65,66^ On the other hand, some studies were not able to identify an influence of antipsychotic treatment on structural brain alterations in patients.^28,67^ Future studies need to investigate the effect of antipsychotics longitudinally to test whether antipsychotic treatment leads to alterations in the cholinergic system. Nevertheless, our findings indicate that the acute state of the first psychotic episode might induce a temporary increase in posterior BFCN-VBM independent of medication.

The observed MRI-based changes might be caused by several nonstructural factors.^51^ As mentioned above, brain water content determines tissue contrast in MRI,^68^ and a recent study has shown increased whole-brain extracellular water volume in FEP.^69^ Thus, the stronger signal may reflect increased water content rather than changes in cell tissue. However, Lawrie suggested that psychosis leads to increased brain volume, and antipsychotic treatment might normalize these changes.^70^ Accordingly, the increased signal in FEP-unmedicated might reflect larger volumes, while in FEP-medicated, changes in lipid content that are affected by antipsychotic treatment result in a decreased MRI signal. To sum up, the findings of larger VBM could reflect alterations in brain structure along with changes in water content, lipid levels, and medication effects.

Differences in posterior BFCN-VBM were only marginally influenced by global GM. Crucially, global GM-VBM in FEP did not differ significantly from that of HC. Global GM reductions have been reported in both ARMS and FEP by some studies^71,72^ but not by others.^73–75^ Based on our data, we suggest that larger posterior BFCN-VBM in FEP reflect a relative specific alteration that is largely independent of the average global GM-VBM.

Our prior research demonstrated lower posterior BFCN-VBM in SCZ.^28^ However, posterior BFCN-VBM in this SCZ sample was not altered. Assuming chronic-progressive changes in the BFCN, this contradiction might result from a lower age and illness duration in the present dataset compared to the previous one. On the other hand, antipsychotic medication might also impact alterations in the posterior BFCN. No differences in posterior BFCN-VBM were found in the ARMS group. This might again indicate that alterations only occur with the first psychotic episode.

### Altered BFCN-VBM is Associated With Impaired Cognition

Regarding the clinical relevance of the detected VBM differences, we demonstrated that BFCN changes were specifically correlated with cognitive deficits. We found corresponding correlations with processing speed in SCZ for the anterior BFCN but only at-trend in FEP for the posterior BFCN. This result was independent of both the cognitive test used and age. This relation seems disorder-specific, as we did not find a corresponding relation in HC, suggesting that the conditions of the disorder are necessary for such a relationship. Furthermore, the association between BFCN and cognitive deficits was symptom-specific, as we did not find a corresponding association for negative or positive symptoms, indicating the special role of BFCN for cognitive deficits in the schizophrenia spectrum. This is in contrast to studies focused on synaptic transmission: evidence demonstrated that pharmacological intervention of the cholinergic system affects not only cognitive but also the psychotic and negative symptoms.^27^

Remarkably, we observed a negative effect of the anticholinergic-burden of medication on the link between BFCN and cognitive performance in SCZ. The anticholinergic-burden influenced the correlation between larger posterior BFCN-VBM and cognitive impairments in FEP. Additionally, it negatively affected lower anterior BFCN-VBM in long-term treated SCZ. Accordingly, recent studies suggest a correlation between the anticholinergic-burden of medication and patients’ cognitive deficits.^76,77^ While it is clear that antipsychotic medication worsens cognitive functioning via anticholinergic effects, our results indicate that, with chronic usage, these anticholinergic effects could also lead to neurobiological changes in the BFCN, which are likewise associated with cognitive symptoms. Future studies are needed to evaluate whether the impact of anticholinergic effects on cognition is brought about by structural changes observed in the BFCN.

## Limitations

In this study, BFCN structure was not measured directly, as volumetric MRI does not solely reflect brain volume. The integrity of the cholinergic system was derived from stereotactic mapping, which is only an indirect marker of cholinergic neurons in the basal forebrain. We used a consensus map based on all available maps of the BFCN, which contains subdivisions based on functional parcellation.^18^ The prior maps were derived from combined postmortem MRI and histology.^38–40^ Previous studies demonstrated that these maps of the BFCN are reliable tools for accurate segmentation of the BFCN.^48,78^ Furthermore, we did not measure individual disorder progression trajectories in this cross-sectional study design. Comparisons across the whole dataset regarding clinical and cognitive measurements were not possible due to the distinct test batteries used across the four sites. Furthermore, our result regarding associations between BFCN-VBM and cognitive scores warrants caution as we did not correct for multiple comparisons. For a detailed description, see Supplemental Discussion.

## Conclusion

We demonstrated a complex and non-linear pattern of posterior and anterior BFCN alterations across the schizophrenia spectrum. BFCN alterations were influenced by medication, including its anticholinergic burden, and specifically associated with patients’ cognitive deficits.

## Supplementary Material

sbad118_suppl_Supplementary_Material
