# Supplementary material for: Basal-Forebrain Cholinergic Nuclei Alterations are Associated With Medication and Cognitive Deficits Across the Schizophrenia Spectrum
Source: Schizophr Bull. 2023 Aug 22;49(6):1530–41. doi: 10.1093/schbul/sbad118 (PMC10686329; doi:10.1093/schbul/sbad118)
Supplement: sbad118_suppl_Supplementary_Material [file sbad118_suppl_supplementary_material.pdf]

Supplemental Material for

Distinct changes in basal forebrain cholinergic nuclei link with cognitive deficits across the schizophrenia spectrum

By Schulz et al.

Table of Contents

Supplementary Methods....2
- Participants....2
  - The Munich cohort....2
  - MRI data acquisition in the Munich cohort....3
  - The COBRE cohort....3
  - MRI data acquisition in the COBRE cohort....4
  - The Zurich cohort....4
  - MRI data acquisition in the Zurich cohort....5
  - The Basel cohort....5
  - MRI data acquisition in the Basel cohort....6
- Assessment of cognitive deficits....6
- Multi-site harmonization across scanners....6
- TIV normalization....7
- Statistical analysis....7

Supplemental Results....9
- Control and specificity analyses for lower anterior BFCN-VBM in schizophrenia....9
- Control and specificity analyses for larger posterior BFCN-VBM in first-episode psychosis patients....11
- Control and specificity analyses for the association between anterior BFCN-VBM and cognitive impairment....13
- Control and specificity analyses for the association between posterior BFCN-VBM and cognitive impairment....15
- Limitations....16

Supplemental Tables....18

Supplemental Figures....19

References....28

Supplementary Methods

Participants

In this cross-sectional, retrospective, case-control, multi-site structural magnetic resonance (MRI)-based volumetry study, datasets from four different research centers were included, comprising healthy controls (HC) and three different groups of the schizophrenia spectrum: individuals at clinical high risk for psychosis (ARMS), first-episode psychosis (FEP), and established schizophrenia (SCZ). A subset of the ARMS cohort later transited to psychosis. The FEP group was further divided into an unmedicated and a medicated subgroup based on antipsychotic drug use (Table 1). Due to low image quality after preprocessing, and missing demographic information (i.e., medication, illness duration), 5 ARMS, 6 FEP, and 6 SCZ were excluded. Quality control was based on IQR output from SPM12. Images with a IQR of 5 standard derivations below and above the mean IQR were excluded.

The Munich cohort

26 SCZ (age range: 23-65 years; mean: 42.84±11.38 years) and 24 age- and sex-matched HC (age range: 25-62 years; mean: 38.54±11.63 years) were recruited from the Department of Psychiatry of Klinikum rechts der Isar, Munich.[1–3] 2 subjects had to be excluded. The patients were diagnosed according to DSM-IV–TR criteria and were in symptomatic remission of psychotic symptoms, evaluated by the criteria of Andreasen.[4,5] Patients' antipsychotic medication was kept stable for at least two weeks prior to the scan. Exclusion criteria for HC were history of Axis I disorder, substance abuse, and first-degree relatives with a history of psychosis. Substance abuse was eliminated via urine-screening or clinical interview. Psychotic and

negative symptoms were evaluated with the Positive and Negative Syndrome Scale (PANSS).[6] This study was approved by the Ethics Review Board of our institution and all participants gave written informed consent.

MRI data acquisition in the Munich cohort

All participants were scanned in an MR-scanner at the Klinikum Rechts der Isar, Munich, Germany. Data acquisition was performed on a hybrid whole-body mMR Biograph PET/MRI scanner (Siemens-Healthineers, Erlangen, Germany), with a vendor-supplied 12-channel phase-array coil. Anatomical data of the whole brain were collected using a T1-weighted MPRAGE sequence with TR/TE/flip angle: 2300 ms/2.98 ms/9°; 160 slices (gap 0.5 mm) covering the whole brain; FoV: 256 mm; matrix size: 256×256; voxel-size: 1×1×1mm3.

The COBRE cohort

The dataset of 72 SCZ meeting DSM-IV-TR criteria[5] (age range: 18-65 years; mean: 38.16±13.89 years) and 73 HC (age range: 18-65 years; mean: 35.60±11.50 years) was derived from the COBRE dataset. Two subjects had to be excluded. Antipsychotic medication was kept stable for minimum four weeks before the study. HC had no history of DSM-IV Axis I disorders, or psychosis in any first-degree relatives. Substance abuse was eliminated via urine-screening. Psychotic and negative symptoms were measured by PANSS.[6] Participants completed their written informed consent.

MRI data acquisition in the COBRE cohort

Data acquisition was performed on a 3 T Siemens Trio MRI scanner with a 12-channel radio frequency coil. Anatomical data of the whole brain were collected using a T1-weighted MPRAGE sequence with TR/TE/TI: 2530/(1.64, 3.5, 5.36, 7.22, 9.08)/900ms, flip angle: 7°, FoV: 256, matrix size: 256×256, voxel-size: 1×1×1mm3. See http://fcon_1000.projects.nitrc.org/indi/retro/cobre.html for more information.

The Zurich cohort

26 FEP (age range: 18-48 years; mean: 24.35±6.89 years), 48 SCZ (age range: 19-49 years; mean: 32.96±7.92 years), and 28 HC (age range: 18-55 years; mean: 32.54±9.15 years) were derived from previous studies.[7–9] Two subjects had to be excluded. FEP were defined as patients with a clinical diagnosis of brief psychotic disorder, schizophreniform disorder or first-episode schizophrenia using Mini-International Neuropsychiatric Interview for DSM-IV (M.I.N.I).[10] FEP with a positive subscale item higher than 5 on PANSS were excluded. SCZ had a clinical diagnosis of schizophrenia. Exclusion criteria for patients were other current DSM-IV axis I disorder, benzodiazepines (>1mg/d lorazepam-equivalent), or extrapyramidal side effects. All FEP and SCZ were treated with antipsychotic medication, the dose was kept stable for minimum two weeks before the study. Exclusion criteria for HC were psychiatric disorders, history of psychiatric disorders, and substance abuse. In schizophrenia, psychotic and negative symptoms were measured by PANSS.[6]

MRI data acquisition in the Zurich cohort

Data acquisition was performed on a Philips Achieva 3.0 T magnetic resonance scanner with a 32-channel SENSE head coil at the MR-Zentrum of the Psychiatric Hospital, University of Zurich. Anatomical data of the whole brain were collected using an ultra-fast gradient echo T1-weighted sequence with TR/TE/flip angle: 8.4ms/3.8ms/8°; 160 slices covering the whole brain; matrix size: 240×240; voxel-size: 1×1×1mm3.

The Basel cohort

73 ARMS (age range: 18-39 years; mean: 24.68±5.12 years), 78 FEP (age range: 18-47 years; mean: 27.51±7.26years), and 44 HC (age range: 19-39 years; mean: 25.52±4.22 years) from two different datasets published by Schmidt and colleagues and Smieskova and colleagues were included in this study.[11,12] 11 subjects had to be excluded.

ARMS were defined as individuals meeting the following inclusion criteria based on the Basel Screening Instrument for Psychosis:[13] "attenuated" psychotic symptoms, brief limited intermittent psychotic symptoms, or a first-degree relative with psychotic disorder and a marked decline in social functioning. After a clinical follow up of 33.3 month, 15 individuals had transited to psychosis. FEP was diagnosed based on the operational criteria for first-episode psychosis according to the ICD-10 or DSM-IV,[14] but not yet for schizophrenia.[15] Specifically, patients' scores on the BPRS were 4 or above on the hallucination item or 5 or above on the unusual thought content, suspiciousness or conceptual disorganization items.[15] 27 FEP were unmedicated, while 45 were treated with antipsychotics. Exclusion criteria for HC were psychiatric

disorders or history of psychiatric disorders, head trauma, neurological illness, serious medical illness, substance abuse, and psychiatric disorder in family history. The Scale for the Assessment of Negative Symptoms (SANS) was used to assess the severity of negative symptoms.[4]

MRI data acquisition in the Basel cohort

Data acquisition was performed on a 3T magnetic resonance imaging scanner (Magnetom Verio, Siemens Healthcare, Erlangen, Germany) at the Basel University Hospital. Anatomical data of the whole brain were collected using a T1-weighted MPRAGE sequence with TR/TE/flip angle: 2000 ms/3.4 ms/8°; 160 slices covering the whole brain; FoV: 176 mm; matrix size: 256×256; voxel-size: 1×1×1mm3.

Assessment of cognitive deficits

For control and specificity analyses the Symbol-coding Task (SCT) of the Brief Assessment of Cognition in Schizophrenia, and Multiple-choice vocabulary intelligence test Part B (MWT-B) were investigated. Both are paper-pencil-based metrics. SCT evaluates attention and processing speed,[16] whereas MWT-B evaluates verbal intelligence.[17]

Multi-site harmonization across scanners

As the present study combined data from four distinct acquisition sites, we controlled for the scanner effect using the open-source toolbox neuroCombat developed by Fortin et al.[18] Total intracranial volume (TIV), anterior, and posterior BFCN-VBM

were harmonized using Empirical Bayes. Sex, age, group, and TIV (for BFCN-VBM) were used as covariates of no interest. For more information, see: https://github.com/Jfortin1/neuroCombat.

TIV normalization

To correct for individual differences in head size, BFCN- and gray matter (GM)-VBM were scaled to TIV by dividing each ROI multiplied by 10^6 (and 10^3 for global GM, respectively) by TIV, as previously described.[19]

Statistical analysis

Specificity analysis for BFCN-VBM. To test whether data were normally distributed D'Agostino's K-squared test was used. Pearson and Spearman correlation analyses were used to investigate the putative association between BFCN-VBM and anticholinergic-burden of medication and antipsychotic medication in the patient groups showing alterations. Anticholinergic-burden of medication was measured using the anticholinergic-burden score (ACB; see: http://www.acbcalc.com),[20] antipsychotic medication was calculated based on chlorpromazine equivalents (CPZ).[21] Only medicated patients were included in the medication association analysis. The specificity of altered BFCN-VBM relative to changes in global GM was examined using ANCOVA across the spectrum, while controlling for the influence of global GM. To test whether age influences and moderates changes in BFCN-VBM, correlation analysis was performed between BFCN-VBM and age in both HC and patients, followed by a moderation analysis using the PROCESS package in SPSS https://www.processmacro.org/download.html. The effect of illness duration on

BFCN-VBM in SCZ was investigated using Spearman correlation, and multiple regression analysis with interaction with anterior BFCN-VBM as dependent variable and age and illness duration as independent variables.

Specificity analysis for altered BFCN-VBM and cognitive deficits. To control for the possible influence of ACB on the association between BFCN-VBM and cognitive deficits, we conducted partial correlation analysis with ACB scores as covariates of no interest. The specificity of cognitive symptoms associated with BFCN-VBM was investigated using correlation analysis with positive and negative PANSS and SANS scales, respectively, instead of cognitive scores, in patients.

Supplemental Results

Control and specificity analyses for lower anterior BFCN-VBM in schizophrenia

First, the influence of medication was tested by correlating the lower anterior BFCN-VBM with both anticholinergic-burden of medication, measured by ACB score, and with antipsychotic medication, measured by CPZ, respectively, in SCZ. The ACB score was significantly negatively correlated with lower anterior BFCN-VBM (rho=-0.22, p=0.009), suggesting that a higher level of anticholinergic-burden of medication is relevant for lower anterior BFCN-VBM in SCZ (Figure S1A). To evaluate the influence of anticholinergic burden of medication, ACB was used as an additional covariate in the ANCOVA model of anterior BFCN changes across the SCZ spectrum. Anterior BFCN-VBM remained at trend significant when controlling for ACB (F4,469=2.50, p=0.06). However, Dunnett's post hoc did not reveal significant differences between patient groups and HC. No significant correlation was found between anterior BFCN-VBM and CPZ (rho=-0.09, p=0.29), indicating that it is unlikely that current antipsychotic medication influences anterior BFCN-VBM of SCZ. Next, changes of anterior BFCN-VBM were compared with changes in the general amount of global GM in SCZ, which might contribute to BFCN changes. Differences in TIV-normalized global GM-VBM between the four groups were tested by the use of ANCOVA. We found significant differences in TIV-normalized global GM across the schizophrenia spectrum ($F_{3,470}$=5.87, p=0.01). Specifically, using Dunnett's' post hoc, global GM-VBM was lower in SCZ compared to HC (Figure S1B). Then, global GM-VBM was used as an additional covariate in the above-mentioned ANCOVA model of anterior BFCN changes across the spectrum to examine the potential

relevance of global GM variations on the group differences observed in anterior BFCN-VBM. *G*lobal GM-VBM changes affected our ANCOVA result, which remained only at-trend significant when controlling for global GM-VBM ($F_{3,469}$=2.07, p=0.10), suggesting that lower anterior BFCN-VBM in SCZ is not independent from global GM-VBM changes. To disentangle the effect of global GM and anticholinergic burden of medication, a partial correlation analysis was conducted between ACB and anterior BFCN-VBM in SCZ with GM-VBM as covariate-of-no-interest. When controlling for global GM-VBM, the correlation between ACB and anterior BFCN-VBM did not remain significant (rho=-0.12, p=0.15), indicating that the association between anterior BFCN-VBM and ACB is not independent of global GM-VBM. Furthermore, we found that ACB was also linked to global GM-VBM in SCZ (rho=-0.23, p=0.007), suggesting an influence of anticholinergic-burden of medication on global GM differences.

Next, we tested whether the lower anterior BFCN-VBM in SCZ is influenced by age. Previous studies demonstrated that age influences BFCN volumes.[19,22] It is unclear whether this is also the case in SCZ and whether age might modify the disorder effect on BFCN-VBM. First, by using correlation analysis, we verified a negative correlation between age and anterior BFCN-VBM in both HC (rho=-0.41, p<0.001) and SCZ (rho=-0.44, p<0.001), respectively. This result suggests that age influences anterior BFCN-VBM not only in HC but also in SCZ (Figure S1C). Then, moderation analysis was used to test whether age moderates the disorder effect (factor disorder with levels HC and SCZ) on anterior BFCN-VBM. The analysis showed no moderation of age on the relationship between disorder and anterior BFCN-VBM

(ΔR^2=1.6%, $F_{1,306}$=0.60, p=0.44), indicating that lower anterior BFCN-VBM in SCZ do not interact with age.

Finally, we asked whether illness duration might modify the disorder effect on anterior BFCN-VBM. To test this, one should remember that age and illness duration are highly correlated (in our case: rho=0.74, p<0.001). Therefore, any test of illness duration effects has to account for age effects too. First, correlation analysis revealed a significant association between anterior BFCN-VBM and illness duration in SCZ (rho=-0.28, p<0.001; Figure S1D). Restricted to SCZ, we performed a multiple regression analysis with interaction with anterior BFCN-VBM as dependent variable and age and illness duration as independent variables. This analysis demonstrated that age and illness duration affect anterior BFCN-VBM in SCZ ($F_{3,136}$=14.01, p<0.001, R^2=0.24). We found again (as above) a main effect of age on anterior BFCN-VBM (p=0.003) but no significant effects for both illness duration (p=0.08) and its interaction with age (p=0.10). This result indicates that illness duration is at least at-trend independent from changes in global GM-VBM beyond age in SCZ. Since age does not appear to significantly modify the effect of the disorder on anterior BFCN-VBM, our result provides indirect support for the model that illness duration is unlikely to modify the disorder effect on BFCN-VBM in SCZ.

Control and specificity analyses for larger posterior BFCN-VBM in first-episode psychosis patients

First, the potential effect of medication on larger posterior BFCN-VBM in FEP was assessed by the use of correlation analysis (Figure S2A). ACB, was not associated with larger posterior BFCN-VBM (rho=-0.07, p=0.56), suggesting that anticholinergic-

burden of medication do not affect posterior BFCN-VBM. Similarly, antipsychotic mediation, CPZ, did not correlate with posterior BFCN-VBM in FEP (rho=0.11, p=0.35), suggesting no influence of current antipsychotic medication on BFCN-VBM changes. Furthermore, posterior BFCN-VBM differs between the groups ($F_{2,262}$=7.15, p=0.001) and is larger in FEP-unmedicated compared to HC (p<0.001).

This result indicates on the one hand, that it is unlikely that larger posterior BFCN-VBM in FEP is driven by antipsychotic medication, and on the other hand, that first episode psychosis might be associated with BFCN-VBM increases. Interestingly, no significant difference in posterior BFCN-VBM was found between HC and FEP-medicated, suggesting that treatment with antipsychotic medication might contribute to normalize larger posterior BFCN-VBM in FEP.

Next, we asked whether larger posterior BFCN-VBM in FEP was influenced by global GM changes. When controlling for global GM-VBM, posterior BFCN-VBM differed at-trend across the schizophrenia spectrum ($F_{3,469}$=2.18, p=0.09; Figure S2B). Additionally, there were no differences in global GM-VBM between HC and FEP, suggesting that larger posterior BFCN-VBM in FEP was not influenced by global GM-VBM changes. To analyze this aspect further, we performed ANCOVA for the two groups (controlling for age, sex, and global GM-VBM). We found a significant difference between the groups ($F_{1,262}$=8.59, p=0.04), with a larger volume in FEP compared to HC (p=0.004), using Dunnett's post hoc test.

To test whether age affects the group difference in posterior BFCN-VBM correlation analysis was used in the HC and FEP groups (Figure S2C). Significant negative correlations between posterior BFCN-VBM and age were found in HC (rho=-0.45, p<0.001), but not in FEP (rho=-0.15, p=0.14). Identical to the approach in anterior

BFCN-VBM, moderation analysis for HC and FEP was performed. We found an at-trend moderation effect of age on the effect of group on posterior BFCN-VBM (ΔR^2=8.9%, $F_{1,263}$=3.10, p=0.08), suggesting that that larger posterior BFCN-VBM in FEP might be driven by interactions between age and psychosis.

Control and specificity analyses for the association between anterior BFCN-VBM and cognitive impairment

First, the effect of anticholinergic-burden of medication on the association between anterior BFCN-VBM and TMT-A was studied using nonparametric partial correlation analysis. The correlation remained significant after controlling for ACB (rho=-0.22, p=0.04), suggesting that anticholinergic-burden of medication do not influence the link between anterior BFCN-VBM and TMT-A.

To investigate whether additional variables such as ACB, sex, and age influenced the relationship between lower anterior BFCN-VBM and TMT-A scores, we computed multiple regression analysis. The overall regression model was significant (R^2=0.13, $F_{81,4}$=4.19, p=0.004). We found that age significantly predicted TMT-A (beta=0.49, p=0.015). ACB, sex, and anterior BFCN-VBM had no significant effect on TMT-A. However, as the link between TMT-A and age is well-known,[23] and age was significantly correlated both with TMT-A (rho =0.42, p<0.001) and anterior BFCN-VBM (rho=-0.44, p<0.001), the latter indicating high collinearity between two predictors. Therefore, multiple regression is not the ideal method to investigate the predictions of TMT-A. We used hierarchical multiple regression with 4 blocks to investigate the effects of each variable on TMT-A (Table S1). The hierarchical multiple regression demonstrated that anterior BFCN-VBM predicts TMT-A values,

as long as age is not included in the model. However, this is not surprising as age is highly collinear with anterior BFCN.

To ensure that the link between anterior BFCN-VBM and cognitive deficit does not depend on the test used for assessing cognitive performance, we investigated the relation between anterior BFCN-VBM and SCT as an alternative measure of cognitive performance with focus on attention and processing speed. ANOVA demonstrated that SCZ had significantly lower SCT scores compared to HC ($F_{178,1}$=38.67, p<0.001). Correlation analysis demonstrated a positive correlation with SCT (rho=0.36, p<0.001) indicating that lower anterior BFCN-VBM in SCZ is associated with lower cognitive test performance, independent of the applied cognitive testing procedure (Figure S3A).

Next, the specificity of the association of lower anterior BFCN-VBM with cognitive deficit in SCZ was examined by correlating the cognitive scores with the anterior BFCN-VBM of HC (Figure S3B). No association was detected between anterior BFCN-VBM and TMT-A (r=-0.11, p=0.30). These findings suggest that the association between lower anterior BFCN-VBM and cognitive functioning is specific to SCZ.

To test for specificity of the association between BFCN-VBM and cognitive performance with respect to other symptom dimensions, anterior BFCN-VBM was correlated with positive and negative PANSS scales, respectively, representing psychotic and negative symptoms of SCZ (Figure S3C). No associations were found between lower anterior BFCN-VBM and PANSS positive (rho=-0.08, p=0.37) or negative (rho=-0.02, p=0.80), respectively, indicating that these symptoms do not

link with the lower anterior BFCN-VBM in SCZ. In other words, lower anterior BFCN-VBM appear to link specifically only with cognitive deficits in SCZ.

Control and specificity analyses for the association between posterior BFCN-VBM and cognitive impairment

First, to test whether the association between posterior BFCN-VBM and Phonemic Fluency was influenced by anticholinergic-burden of medication, partial correlation analysis between posterior BFCN-VBM and Phonemic Fluency scores was computed. After controlling for ACB, no association between posterior BFCN-VBM and Phonemic Fluency was detectable (rho=-0.07, p=0.74), suggesting an influence of anticholinergic-burden of medication on this association (Figure S4A).

Next, to ensure that the link between posterior BFCN-VBM and cognitive deficit does not depend on the test used for assessing cognitive performance, we investigated the relation between posterior BFCN-VBM and MWT-B score as an alternative measure of cognitive performance. ANOVA demonstrated significantly reduced performance in MWT-B in FEP compared to HC ($F_{111,1}$=4.39, p=0.038). Pearson's correlation analysis demonstrated that posterior BFCN-VBM correlated with MWT-B score at-trend (r=-0.20, p=0.10), suggesting that the relevance of posterior BFCN-VBM increases for impaired cognitive performance does not depend – at least at-trend - on the used cognitive test.

Furthermore to test for specificity with respect to group, associations with cognitive impairment measured by Phonemic Fluency and posterior BFCN-VBM were also tested in the other groups of the spectrum and HC using correlation analysis (Figure S4B). The posterior BFCN-VBM showed no correlation in HC (r=0.02 p=0.82) nor in

ARMS (r=-0.29, p=0.16). However, a link was found between posterior BFCN-VBM and Phonemic Fluency in SCZ, which was at-trend to significance (r=0.17, p=0.09). This finding suggests that posterior BFCN-VBM of clinically more affected groups of the spectrum including FEP might be relevant for cognitive performance.

Finally, to test for specificity of the association between posterior BFCN-VBM and cognitive performance with respect to other symptom dimensions, the correlation of posterior BFCN-VBM with negative and psychotic symptoms, respectively, was investigated in FEP (Figure S4C). No associations were found between posterior BFCN and psychotic symptoms (r=-0.28, p=0.17), measured by PANSS positive, and negative symptoms (rho=-0.01, p=0.95), measured by SANS scale. These results suggest that neither psychotic nor negative symptoms link with posterior BFCN-VBM in FEP.

Limitations

The present study has several limitations. First, regarding the measurement, so-called volumetric MRI does not directly measure brain volume , making clear-cut and consistent interpretations of MRI-based changes difficult.[24,25] Second, the integrity of the cholinergic system is assessed indirectly by MRI-based techniques. The volumetric measurements of the BFCN were determined by stereotactic mapping of the BFCN, and thus non-cholinergic neuronal populations interacting with cholinergic neurons in the BF are included within the ROI.[26,27] A recent study reported that stereotactic mapping of nucleus basalis Meynert generates deviating results from post-mortem findings.[28] While our results indicate that structural changes in the basal forebrain are relevant for patients' cognitive deficits, we cannot demonstrate that the

functional outcome solely relies on changes in cholinergic neurons. Functional imaging studies might disentangle the functional relevance of BFCN alterations along the course of schizophrenia to a greater extent. Third, the present study examined cross-sectional data, thus, we cannot derive individual disorder progression trajectories. Longitudinal studies might be employed to provide better insights into the effect of psychosis, long-term treatment, and chronicity on structural changes in the cholinergic system. Fourth, distinct clinical and cognitive variables were acquired across the different sites, which engraves comparisons of associations across the whole dataset. Therefore, findings should be evaluated carefully. Finally, the sample size of specific subgroups, namely both at-risk individuals that transited to psychosis and unmedicated first-episode patients, was relatively modest, calling for larger sample size studies in the future.

Supplemental Tables

Table S1. Hierarchical multiple regression with four blocks investigating the effect of anterior BFCN-VBM, ACB, sex, and age on TMT-A in schizophrenia. ACB anticholinergic-burden of medication, BFCN basal-forebrain cholinergic nuclei.

Model	Variables	beta	R^2	Overall Model p
1	anterior BFCN-VBM	-0.16*	0.076	0.006
2	anterior BFCN-VBM	-0.13*	0.087	0.008
	ACB	1.83		
3	anterior BFCN-VBM	-0.13*	0.076	0.02
	ACB	1.83		
	sex	0.05		
4	anterior BFCN-VBM	-0.03	0.13	0.004
	ACB	1.99		
	sex	1.55		
	age	0.49*		

Supplemental Figures

A. Altered anterior BFCN and medication:

i. Anticholinergic medication

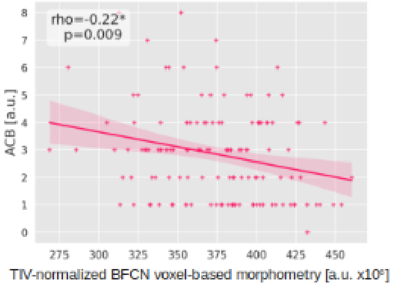

n=135

ii. Antipsychotic medication

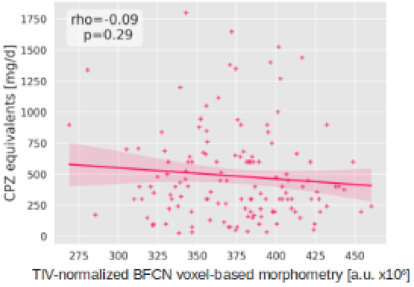

n=132

B. Control for global GM:

i. Voxel-based morphometry of global GM

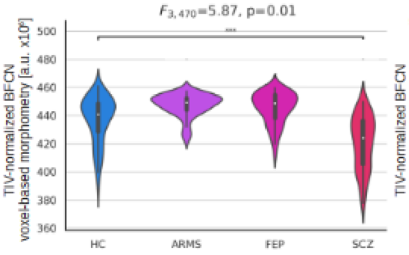

n= 169 68 98 140

ii. Anterior BFCN voxel-based morphometry corrected for global GM

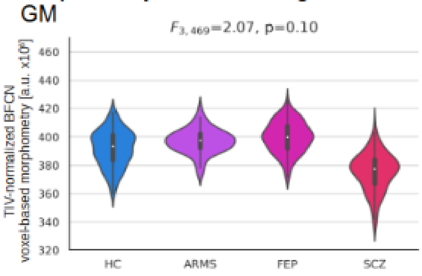

n= 169 68 98 140

C. Anterior BFCN and age:

i. Correlation in HC

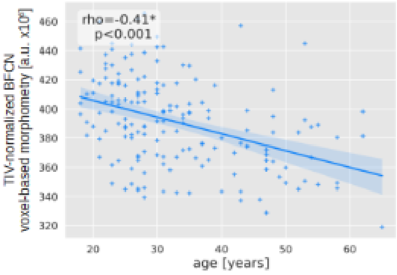

n=169

ii. Correlation in SCZ

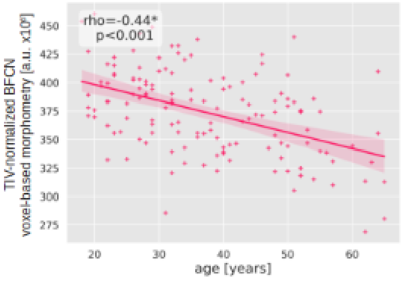

n=140

iii. Moderation analysis

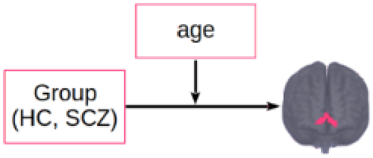

$F_{3,306}$ =28.43, p<0.001
ΔR^2=1,6%, $F_{1,306}$ = 0.60, p=0.44

D. Anterior BFCN and illness duration:

i. Effect of illness duration

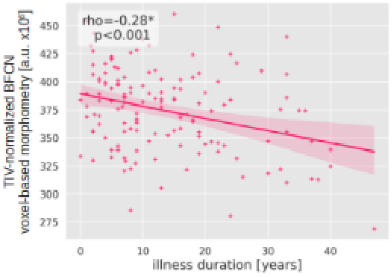

n=140

Figure S1. Control and specificity analyses for lower anterior BFCN-VBM in schizophrenia. (A) Associations between lower anterior BFCN-VBM and medication in SCZ were investigated using Spearman correlation. (i) Plot shows a significant correlation between anterior BFCN-VBM and ACB (rho=-0.22, p=0.009). (ii) No significant correlation was found between anterior BFCN-VBM and CPZ (rho=-0.09, p=0.29). (B) Global GM influences on regional changes between HC, ARMS, FEP, and SCZ were investigated using ANCOVA controlling for age and sex. Dunnett's was used for post-hoc analysis . *<0.05, **<0.01, and ***<0.001. (i) Global GM-VBM differed significantly across the schizophrenia spectrum ($F_{3,470}$=5.87, p=0.01) and was lower in SCZ compared to HC (p<0.001). (ii) Anterior BFCN-VBM were compared between groups when additionally controlling for TIV-normalized global GM. Anterior BFCN-VBM differed at-trend across the schizophrenia spectrum ($F_{3,469}$=2.07, p=0.10) and was lower in SCZ compared to HC (p=0.039). (C) Associations between anterior BFCN-VBM and age were investigated using Spearman correlation. Plot shows a significant correlation between anterior BFCN-VBM and age in (i) HC (rho=-0.41, p<0.001) and (ii) SCZ (rho=-0.44, p<0.001). (iii) Moderation analysis was used to investigate whether the interaction of age and group predicts the group difference in anterior BFCN-VBM. The overall model was significant ($F_{3,306}$=28.43, p<0.001), but the group difference in anterior BFCN-VBM was not moderated by age and group interaction (ΔR^2=1.6%, $F_{1,306}$=0.60, p=0.44). (D) Associations between lower anterior BFCN-VBM and illness duration were investigated using Spearman correlation. Plot shows a significant correlation between anterior BFCN-VBM and illness duration in SCZ (rho=-0.28, p<0.001). ACB anticholinergic-burden of medication, ARMS individuals with a high risk for

psychosis, BFCN basal-forebrain cholinergic nuclei, CPZ chlorpromazine equivalents, FEP patients with first-episode psychosis, GM gray matter, HC healthy controls, SCZ patients with established schizophrenia, TIV total intracranial volume, VBM voxel-based morphometry.

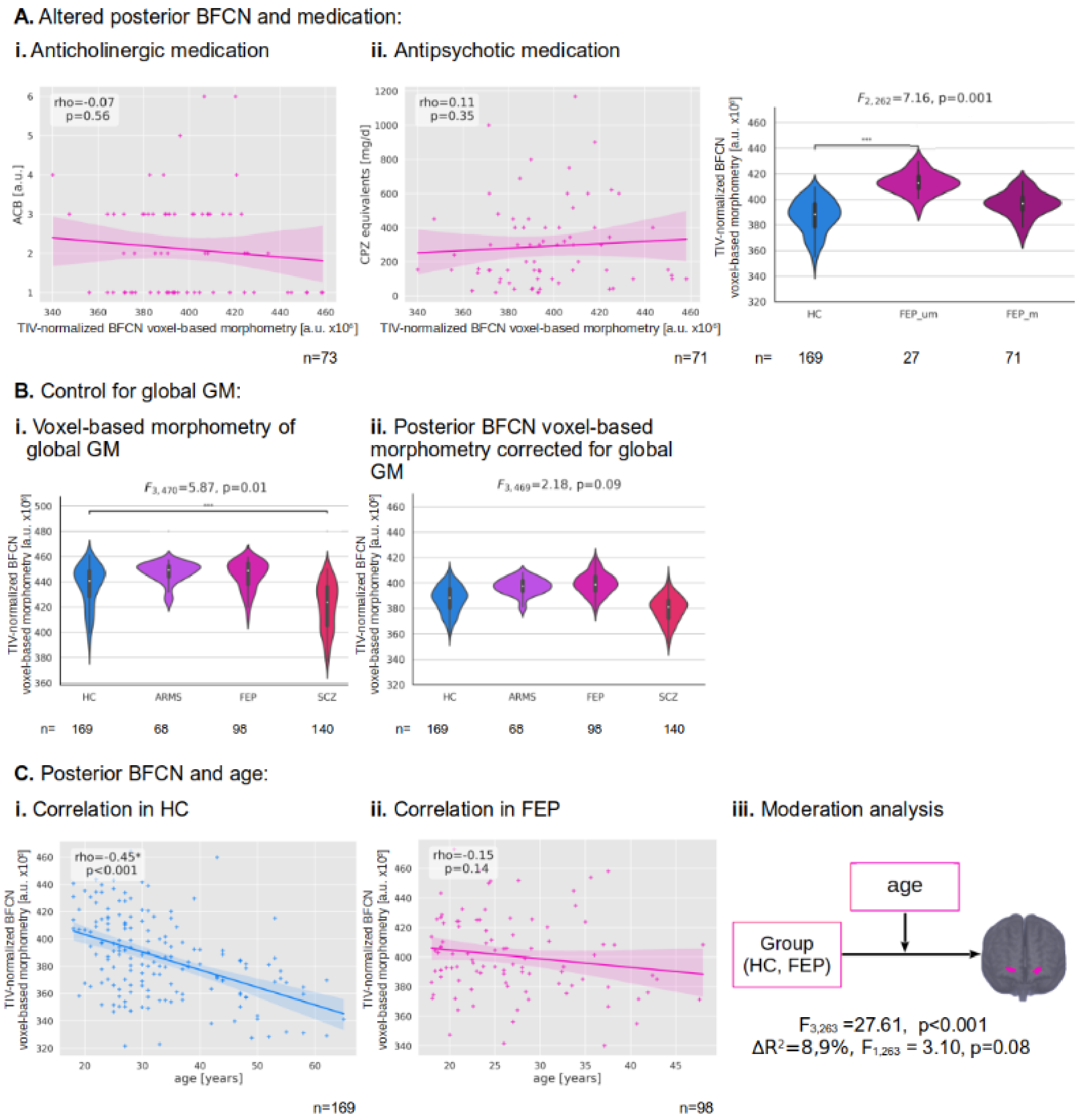

Figure S2. Control and specificity analyses for larger posterior BFCN-VBM in first-episode-psychosis patients. (A) Associations between larger posterior BFCN-VBM and medication in FEP were investigated with Spearman correlation. (i) No significant correlation between posterior BFCN-VBM and ACB was found (rho=-0.07, p=0.56). (ii). No significant correlation between posterior BFCN-VBM and CPZ was found (rho=0.11, p=0.35). (iii) Comparison between HC, FEP-unmedicated, and

FEP-medicated regarding posterior BFCN-VBM was investigated using ANCOVA controlling for age and sex. Dunnett's was used for post-hoc analysis. *<0.05, **<0.01, and ***<0.001. Posterior BFCN-VBM differs between the groups ($F_{2,262}$=7.15, p=0.001) and is larger in FEP-unmedicated compared to HC (p<0.001). (B) Global GM influences on regional changes between HC, ARMS, FEP, and SCZ were investigated using ANCOVA controlling for age and sex. (i) Global GM-VBM differed significantly across the schizophrenia spectrum ($F_{3,470}$=5.87, p=0.01) and was lower in SCZ compared to HC (p<0.001). (ii) Posterior BFCN-VBM were compared between groups when controlling additionally for TIV-normalized global GM-VBM. Posterior BFCN-VBM differed at-trend across the schizophrenia spectrum ($F_{3,469}$=2.18, p=0.09) and was larger in FEP compared to HC (p=0.01). (C) Associations between posterior BFCN-VBM and age in HC and FEP were investigated using Spearman correlation. Plot shows significant correlations between posterior BFCN-VBM and age in (i) HC (rho=-0.45, p<0.001), but not in (ii) FEP (rho=-0.15, p=0.14). (iii) Moderation analysis was used to investigate whether the interaction of age and group predicts the group difference in posterior BFCN-VBM. The overall model was significant ($F_{3,263}$=27.61, p<0.001), but the group difference in posterior BFCN-VBM was not moderated by age and group interaction (ΔR^2 = 8.9%, $F_{1,263}$=3.10, p=0.08). ACB anticholinergic-burden of medication, ARMS individuals with a high risk for psychosis, BFCN basal-forebrain cholinergic nuclei, CPZ chlorpromazine equivalents, FEP patients with first-episode psychosis, GM gray matter, HC healthy controls, SCZ patients with established schizophrenia, TIV total intracranial volume, VBM voxel-based morphometry.

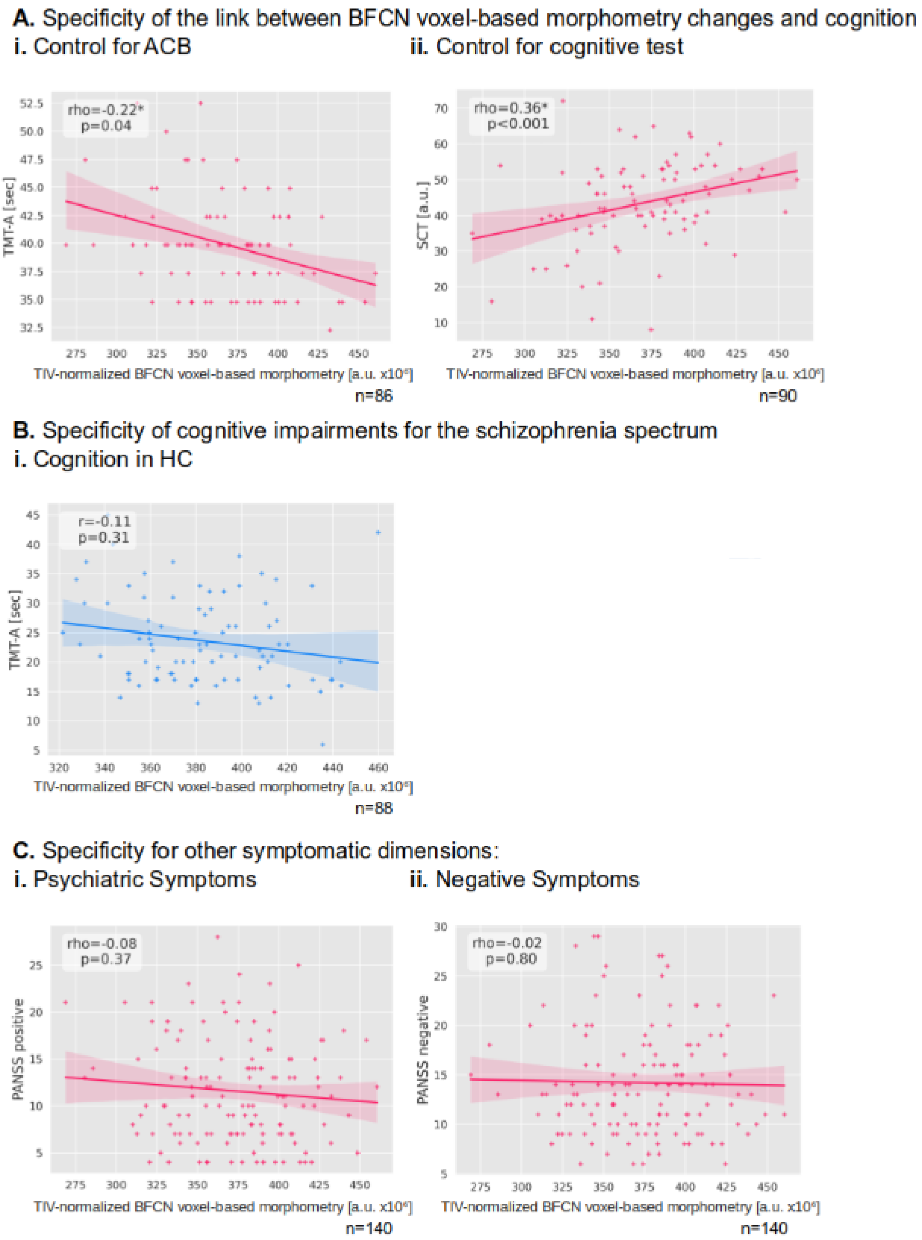

Figure S3. Control and specificity analyses for the association between anterior BFCN-VBM and cognitive impairment. (A) Associations between anterior BFCN-VBM and symptoms were investigated with Spearman correlation. (i) Plot shows significant correlation between anterior BFCN-VBM and TMT-A score when controlling for ACB in SCZ (rho=-0.22, p=0.04). (ii) Plot shows a significant correlation between anterior BFCN-VBM and SCT in SCZ (rho=0.36, p<0.001). (B)

No significant correlation was found between anterior BFCN-VBM and TMT-A score in HC (r=-0.11, p=0.30). (C) Associations between anterior BFCN-VBM and symptoms were investigated with Spearman correlation. In SCZ, no significant correlation was found between anterior BFCN-VBM and neither (i) PANSS positive (rho=-0.08, p=0.37) nor (ii) PANSS negative score (rho=-0.02, p=0.80). ACB anticholinergic-burden of medication, ARMS individuals with a high risk for psychosis, BFCN basal-forebrain cholinergic nuclei, FEP patients with first-episode psychosis, HC healthy controls, PANSS Positive and Negative Syndrome Scale, SCT Symbol-coding Task, SCZ patients with established schizophrenia, TMT-A Trail Making Test Part A, VBM voxel-based morphometry.

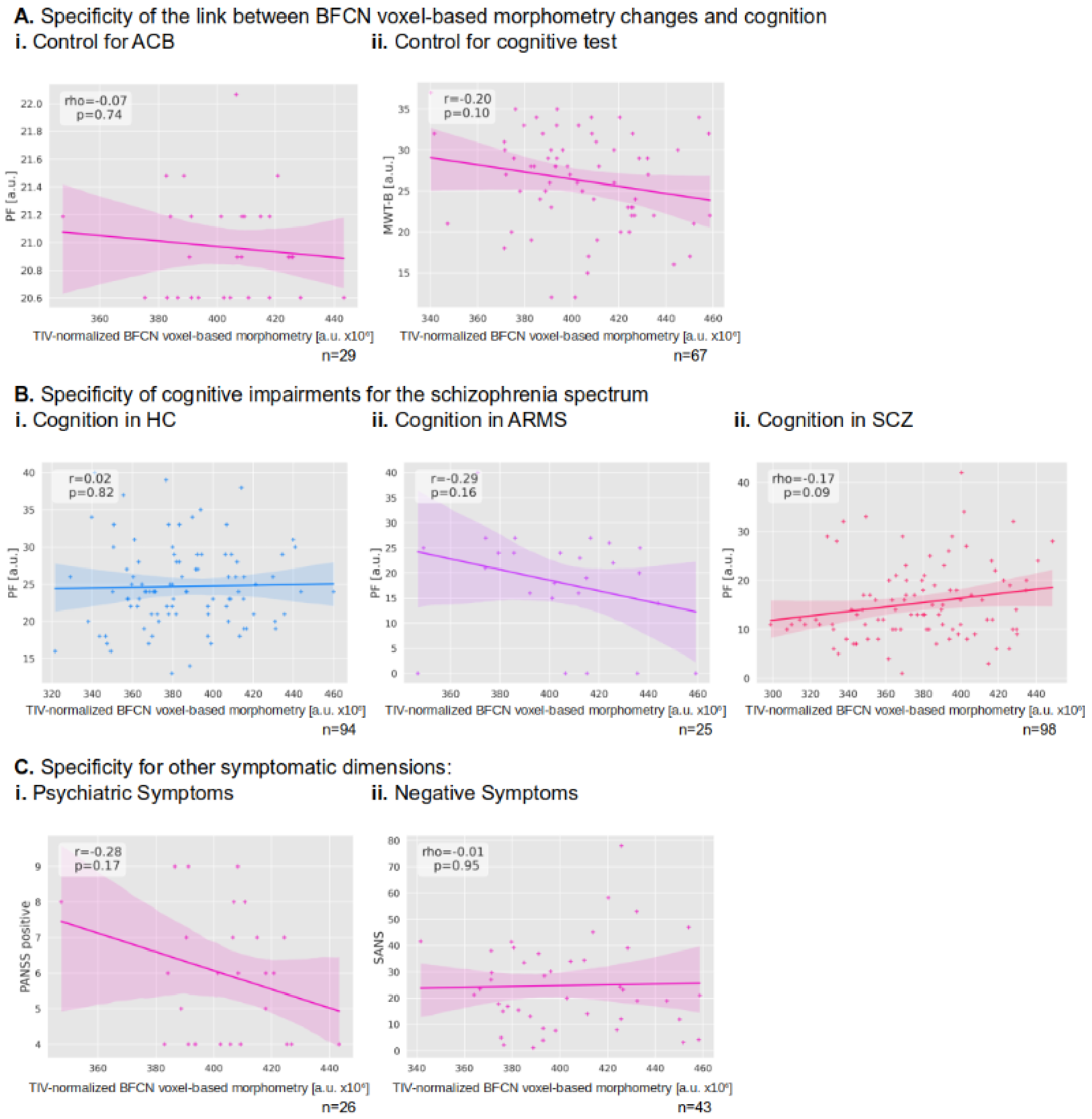

Figure S4: Control and specificity analyses for the association between posterior BFCN-VBM and cognitive impairment. Associations between posterior BFCN-VBM and symptoms were investigated using correlation analysis. (Ai) No significant correlation was found between posterior BFCN-VBM and PF when controlling for ACB in FEP (rho=-0.07, p=0.74). (ii) Plot shows a correlation at-trend to significance between posterior BFCN-VBM and MWT-B in FEP (r=-0.20, p=0.10).

(B) Pearson correlation did not show a significant correlation neither in (i) HC (r=0.02, p=0.82) nor (ii) ARMS (r=-0.29, p=0.16). (iii) No significant correlation between posterior BFCN-VBM and PF was found in SCZ using Spearman correlation (rho=0.17, p=0.09). (Ci) No significant correlation was found between posterior BFCN-VBM and PANSS positive (r=-0.28, p=0.17), and (ii) SANS score (rho=-0.01, p=0.95). ACB anticholinergic-burden of medication, ARMS individuals with a high risk for psychosis, BFCN basal-forebrain cholinergic nuclei, FEP patients with first-episode psychosis, HC healthy controls, MWT-B Multiple-choice vocabulary intelligence test Part B, PANSS Positive and Negative Syndrome Scale, PF Phonetic fluency, SCZ patients with established schizophrenia, TMT-A Trail Making Test Part A, VBM voxel-based morphometry.

References

1. Avram M, Grothe MJ, Meinhold L, et al. Lower cholinergic basal forebrain volumes link with cognitive difficulties in schizophrenia. *Neuropsychopharmacology*. 2021;46(13):2320-2329. doi:10.1038/s41386-021-01070-x

2. Avram M, Brandl F, Cabello J, et al. Reduced striatal dopamine synthesis capacity in patients with schizophrenia during remission of positive symptoms. *Brain*. 2019;142(6):1813-1826. doi:10.1093/brain/awz093

3. Brandl F, Knolle F, Avram M, et al. Negative symptoms, striatal dopamine and model-free reward decision-making in schizophrenia. *Brain*. July 2022:awac268. doi:10.1093/brain/awac268

4. Andreasen NC, Carpenter WT, Kane JM, Lasser RA, Marder SR, Weinberger DR. Remission in schizophrenia: Proposed criteria and rationale for consensus. *Am J Psychiatry*. 2005;162(3):441-449. doi:10.1176/APPI.AJP.162.3.441/ASSET/IMAGES/LARGE/N83T2.JPEG

5. First MB, Spitzer R, Gibbon M, Williams JBW. *Structured Clinical Interview for DSM-IV-TR Axis I Disorders, Research Version, Patient Edition (SCID-I/P)*. New York: Biometrics Research: New York State Psychiatric Institute; 2002.

6. Kay SR, Fiszbein A, Opler LA. The Positive and Negative Syndrome Scale (PANSS) for Schizophrenia. *Schizophr Bull*. 1987;13(2):261-276. doi:10.1093/SCHBUL/13.2.261

7. Kirschner M, Hager OM, Bischof M, et al. Ventral striatal hypoactivation is associated with apathy but not diminished expression in patients with schizophrenia. *J Psychiatry Neurosci*. 2016;41(3):152-161. doi:10.1503/jpn.140383

8. Kirschner M, Hager OM, Muff L, et al. Ventral Striatal Dysfunction and Symptom Expression in Individuals with Schizotypal Personality Traits and Early Psychosis. *Schizophr Bull*. 2018;44(1):147-157. doi:10.1093/schbul/sbw142

9. Kirschner M, Schmidt A, Hodzic-Santor B, et al. Orbitofrontal-Striatal Structural Alterations Linked to Negative Symptoms at Different Stages of the Schizophrenia Spectrum. *Schizophr Bull*. 2021;47(3):849-863. doi:10.1093/schbul/sbaa169

10. Lecrubier Y, Sheehan DV, Weiller E, et al. The Mini International Neuropsychiatric Interview (MINI). A short diagnostic structured interview: reliability and validity according to the CIDI. *Eur Psychiatry*. 1997;12(5):224-231. doi:10.1016/S0924-9338(97)83296-8

11. Schmidt A, Lenz C, Smieskova R, et al. Brain Diffusion Changes in Emerging Psychosis and the Impact of State-Dependent Psychopathology. *NeuroSignals*. 2015;23(1):71-83. doi:10.1159/000442605

12. Smieskova R, Roiser JP, Chaddock CA, et al. Modulation of motivational salience processing during the early stages of psychosis. *Schizophr Res*. 2014;166(1-3):17-23. doi:10.1016/j.schres.2015.04.036

13. Riecher-Rössler A, Pflueger MO, Aston J, et al. Efficacy of using cognitive status in predicting psychosis: a 7-year follow-up. *Biol Psychiatry*. 2009;66(11):1023-1030. doi:10.1016/J.BIOPSYCH.2009.07.020

14. Breitborde NJK, Srihari VH, Woods SW. Review of the operational definition for first-episode psychosis. *Early Interv Psychiatry*. 2009;3(4):259-265. doi:10.1111/J.1751-7893.2009.00148.X

15. Yung AR, McGorry PD. The prodromal phase of first-episode psychosis: past and current conceptualizations. *Schizophr Bull*. 1996;22(2):353-370.

16. Keefe RSE, Goldberg TE, Harvey PD, Gold JM, Poe MP, Coughenour L. The Brief Assessment of Cognition in Schizophrenia: reliability, sensitivity, and comparison with a standard neurocognitive battery. *Schizophr Res*. 2004;68(2-3):283-297. doi:10.1016/J.SCHRES.2003.09.011

17. Lehrl S, Triebig G, Fischer B. Multiple choice vocabulary test MWT as a valid and short test to estimate premorbid intelligence. *Acta Neurol Scand*. 1995;91(5):335-345. doi:10.1111/j.1600-0404.1995.tb07018.x

18. Fortin JP, Cullen N, Sheline YI, et al. Harmonization of cortical thickness measurements across scanners and sites. *NeuroImage*. 2018;167:104-120. doi:10.1016/j.neuroimage.2017.11.024

19. Grothe MJ, Scheef L, Bäuml J, et al. Reduced Cholinergic Basal Forebrain Integrity Links Neonatal Complications and Adult Cognitive Deficits After Premature Birth. *Biol Psychiatry*. 2017;82(2):119-126. doi:10.1016/j.biopsych.2016.12.008

20. Kiesel EK, Hopf YM, Drey M. An anticholinergic burden score for German prescribers: Score development. *BMC Geriatr*. 2018;18(1). doi:10.1186/s12877-018-0929-6

21. Gardner DM, Murphy AL, O'Donnell H, Centorrino F, Baldessarini RJ. International consensus study of antipsychotic dosing. *Am J Psychiatry*. 2010;167(6):686-693. doi:10.1176/APPI.AJP.2009.09060802

22. Grothe M, Heinsen H, Teipel S. Longitudinal measures of cholinergic forebrain atrophy in the transition from healthy aging to Alzheimer's disease. *Neurobiol Aging*. 2013;34(4):1210-1220. doi:10.1016/J.NEUROBIOLAGING.2012.10.018

23. Kennedy KJ. Age effects on Trail Making Test performance. *Percept Mot Skills*. 1981;52(2):671-675. doi:10.2466/pms.1981.52.2.671

24. Weinberger DR, Radulescu E. Structural Magnetic Resonance Imaging All over Again. *JAMA Psychiatry*. 2021;78(1):11-12. doi:10.1001/jamapsychiatry.2020.1941

25. Schulz J, Zimmermann J, Sorg C, Menegaux A, Brandl F. Magnetic resonance imaging of the dopamine system in schizophrenia – A scoping review. *Front Psychiatry*. 2022;0:1682. doi:10.3389/FPSYT.2022.925476

26. Zaborszky L, Hoemke L, Mohlberg H, Schleicher A, Amunts K, Zilles K. Stereotaxic probabilistic maps of the magnocellular cell groups in human basal forebrain. *NeuroImage*. 2008;42(3):1127-1141. doi:10.1016/J.NEUROIMAGE.2008.05.055

27. Yang Y, Wang JZ. From structure to behavior in basolateral amygdala-hippocampus circuits. *Front Neural Circuits*. 2017;11. doi:10.3389/fncir.2017.00086

28. Wang Y, Zhan M, Roebroeck A, De Weerd P, Kashyap S, Roberts MJ. Inconsistencies in atlas-based volumetric measures of the human nucleus basalis of Meynert: A need for high-resolution alternatives. *NeuroImage*. 2022;259:119421. doi:10.1016/J.NEUROIMAGE.2022.119421
